# A Root in Synapsis and the Other One in the Gut Microbiome-Brain Axis: Are the Two Poles of Ketogenic Diet Enough to Challenge Glioblastoma?

**DOI:** 10.3389/fnut.2021.703392

**Published:** 2021-07-22

**Authors:** Liliana Montella, Federica Sarno, Lucia Altucci, Valentina Cioffi, Luigi Sigona, Salvatore Di Colandrea, Stefano De Simone, Alfredo Marinelli, Bianca Arianna Facchini, Ferdinando De Vita, Massimiliano Berretta, Raffaele de Falco, Gaetano Facchini

**Affiliations:** ^1^Medical Oncology Complex Unit, “Santa Maria delle Grazie” Hospital, ASL Napoli 2 Nord, Naples, Italy; ^2^Precision Medicine Department, “Luigi Vanvitelli” University of Campania, Naples, Italy; ^3^Neurosurgery Operative Complex Unit, “Santa Maria delle Grazie” Hospital, ASL Napoli 2 Nord, Naples, Italy; ^4^Department of Emergency and Critical Care, “Santa Maria delle Grazie” Hospital, ASL Napoli 2 Nord, Naples, Italy; ^5^Operative Unit Neuroncology University Federico II, Naples, Italy; ^6^Istituto di Ricerca e Cura a Carattere Scientifico (IRCCS) Neuromed Istituto Neurologico Mediterraneo (INM), Isernia, Italy; ^7^Division of Medical Oncology, Precision Medicine Department, “Luigi Vanvitelli” University of Campania, Naples, Italy; ^8^Department of Clinical and Experimental Medicine, University of Messina, Messina, Italy

**Keywords:** glioblastoma, ketogenic diet, gut-brain axis, microbiota, warburg effect

## Abstract

Glioblastoma is the most frequent and aggressive brain cancer in adults. While precision medicine in oncology has produced remarkable progress in several malignancies, treatment of glioblastoma has still limited available options and a dismal prognosis. After first-line treatment with surgery followed by radiochemotherapy based on the 2005 STUPP trial, no significant therapeutic advancements have been registered. While waiting that genomic characterization moves from a prognostic/predictive value into therapeutic applications, practical and easy-to-use approaches are eagerly awaited. Medical reports on the role of the ketogenic diet in adult neurological disorders and in glioblastoma suggest that nutritional interventions may condition outcomes and be associated with standard therapies. The acceptable macronutrient distribution of daily calories in a regular diet are 45–65% of daily calories from carbohydrates, 20–35% from fats, and 10–35% from protein. Basically, the ketogenic diet follows an approach based on low carbohydrates/high fat intake. In carbohydrates starvation, body energy derives from fat storage which is used to produce ketones and act as glucose surrogates. The ketogenic diet has several effects: metabolic interference with glucose and insulin and IGF-1 pathways, influence on neurotransmission, reduction of oxidative stress and inflammation, direct effect on gene expression through epigenetic mechanisms. Apart from these central effects working at the synapsis level, recent evidence also suggests a role for microbiome and gut-brain axis induced by a ketogenic diet. This review focuses on rationales supporting the ketogenic diet and clinical studies will be reported, looking at future possible perspectives.

## Introduction

All published papers concerning brain tumors and in particular glioblastoma (GBM) almost invariably start with the same dismal outlook made of a bad prognosis and median overall survival ranging between 12 and 15 months. Different from other malignancies characterized by significant advancements in precision medicine and increased application of active medical before local treatments in proposed algorithms, the mainstay of GBM treatment remains radical surgery with preservation of functionally crucial brain areas, followed by radiotherapy and concomitant and adjuvant temozolomide. At GBM recurrence, surgery must be considered again, if feasible, given the lack of effective medical treatments. Few signs of progress have been registered in the GBM landscape after the landmark STUPP trial published more than 15 years ago (1). In this perspective, the publication of the 2016 WHO blue book represents the transition from a morphological to a combined histo-molecular classification of brain tumors. This classification encompasses molecular markers like isocitrate dehydrogenase (IDH)1/IDH2 mutations and 1p/19q codeletion for diffuse gliomas. Such biomarkers have a recognized prognostic and predictive value. Moreover, IDH 1/2 mutations are considered an early step in gliomagenesis, followed by 1p/19q co-deletion which is a hallmark of grade II/III oligodendroglioma. Nearly all 1p/19q codeleted GBMs have an IDH 1/2 mutation. MGMT promoter methylation is also part of this genomic profile, setting up a favorable signature. The precursor cell harboring IDH 1/2 mutations gains additional mutations, involving tp53 and ATRX, and gives rise to grade II/III astrocytoma. This latter can gain Rb1 loss/mutation, CDK4/6 amplification, PDGFRA amplification, or lose CDKN2A and, over the years, becomes IDH-mutated secondary glioblastoma. Primary GBM lacks IDH mutations and 1p/19q codeletion in 95% of the cases, this pattern being considered associated with poor prognosis. The methylation of the MGMT promoter further characterizes GBM with a better prognosis and response to temozolomide. MGMT promoter methylation confers sensitivity to temozolomide and differentiates patients with poor and bad prognosis. A recent meta-analysis confirms initial data and found a median overall survival (OS) of about 14 months as compares to 24 for patients with unmethylated and methylated GBM, respectively. Progression-free survival (PFS) was almost doubled in methylated (10 months) compared to unmethylated patients (about 5 months) (2). However, the irrefutable value of MGMT promoter methylation as a prognostic and predictive marker has not therapeutic reverse: lack of methylation does not translate into changes in therapeutic algorithms to date.

Compared to the available biomarker profile which helps in defining prognosis and predicts response to available treatments, a definite assessment of GBM pathogenesis and identification of key pivotal pathways are still lacking. GBM represents a challenge because of different peculiar issues: the blood-brain barrier is regarded as an edge for drugs, brain tumors are characterized by a natural propensity to local recurrence, heterogeneity concerns different tumor areas and recurrence vs. original tumor. All these aspects condition the limited activity shown by targeted drugs and rationale-designed clinical trials. On date of April 6th, 2021, only 15 recruiting phase III clinical trials are available for GBM patients.^1^

In this gray setting, the search for alternative strategies can easily take place. Integrative medicine and especially restrictive and ketogenic diets have been proposed based on studies on tumor cell metabolism and preclinical models.

In the present paper, after a general overview of GBM genomics and cell metabolism, we deal with the rationales of ketogenic/restricted diet and results reported by clinical studies with speculations on the future of nutritional interventions in this setting.

## GBM Genomics and Bio-Energetic Pathways Interplay

Increasing evidence supports the mutual and uncoupled dependence between oncogenic signaling triggered by growth factors and metabolic reprogramming to support tumor cell growth.

Three major altered pathways have been identified in GBM: (1) receptor tyrosine kinases including EGFR, PDGFR, cMET, PDGFR, Her2, and downstream ras and PI3K/PTEN/AKT/mTOR pathway (90%), (2) deregulations in TP53, MDM2, and MDM4 (around 85%), (3) Rb signaling and cell cycle-related pathways which include cyclins (3). Copy number aberrations are frequently found in GBM and include chromosomes 9 and 10 loss, polysomy in chromosomes 7, 19, 20, focal deletion of CDKN2A/B locus, and amplification of EGFR locus.

Epidermal growth factor receptor (EGFR) has a pivotal role in several tumors and among them lung cancer, breast cancer, and GBM. The EGFR gene is located on chromosome 7p11.2 and encodes a transmembrane protein receptor. The Ligand Binding domain (LBD) derives from the transcription of exons 5–7 and 13–16, while the tyrosine kinase domain derives from exons 18–24 (4). In gliomas, heterogeneous mutations and deletions are grouped on the ligand-binding ectodomain (ECD) of EGFR. The result of this mutation is a constitutively activated receptor in absence of ligand. Almost 50% of the tumors present the mutant EGFRvIII and EGFR single nucleotide variants (SNVs). The variant EGFRVIII is found in 25% of the cases and is associated with a poor prognosis. The activation of EGFR signaling induces increased proliferation and migration of different cell types (5).

**Figure 1:**
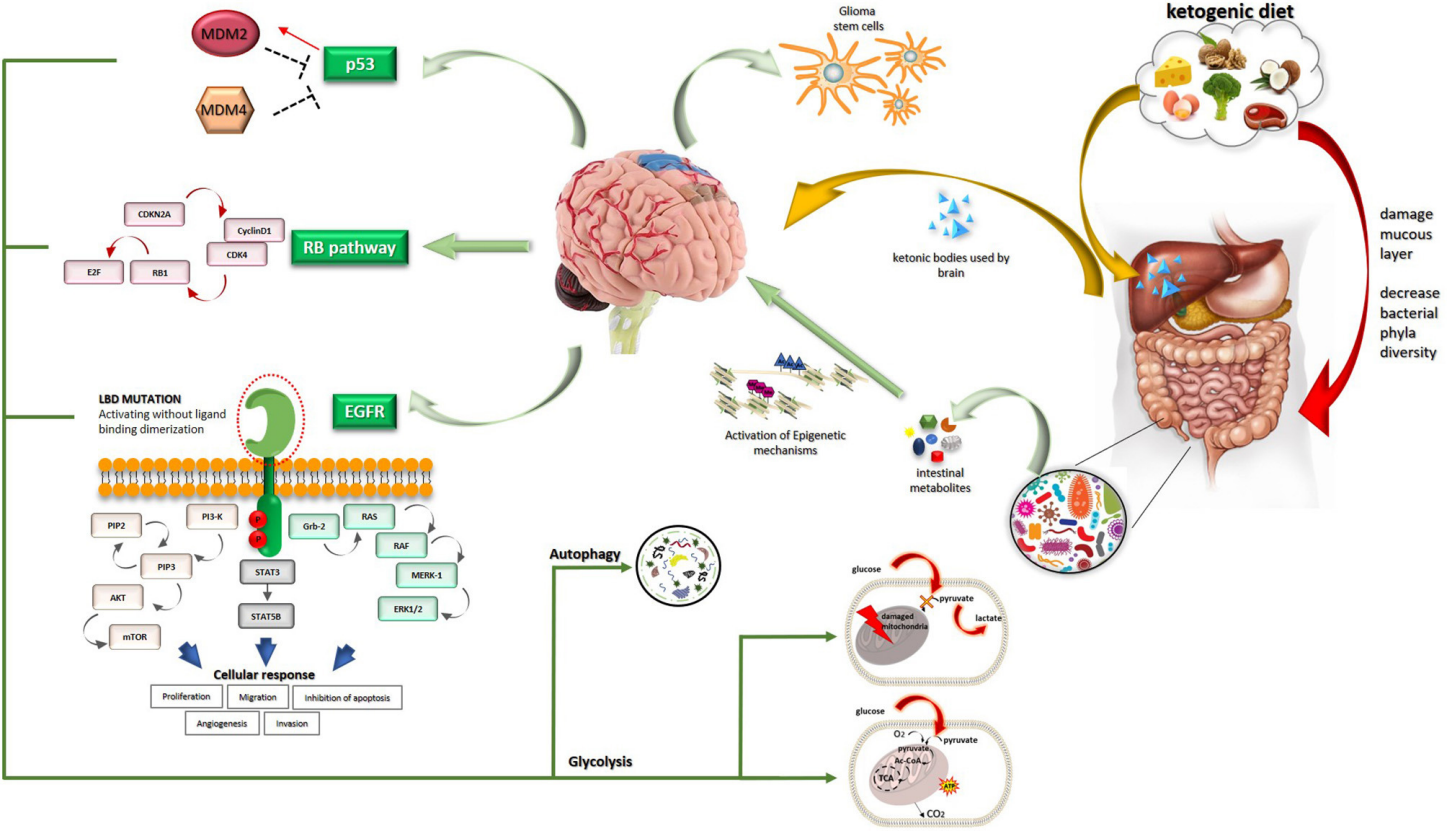
A comprehensive view of oncogenic and metabolic pathways involved in GBM is described.

Oncogenic signallings are strictly connected with bio-energetic pathways (Figure 1). The EGFR plays an important role in the intracellular degradation system called “autophagy” that functions as a scavenger removing damaged organelles, malformed or non-functional proteins (6). Autophagy is a mechanism that can be limited and takes survival advantage to cells or, carried to the extreme, can lead to cell death. This process relies predominantly upon so-called mitochondrial dynamics. Two different processes involving mitochondria can realize in response to cellular stress and produce different results. The first is mitochondrial fusion which allows the cancer cell to reply to increased energy demand and corresponds to a boosted respiratory capacity. In contrast, mitochondrial fission leads to autophagy which provides cellular materials especially in times of deprivation or alternatively leads to apoptosis. Mitochondrial fission has been reported in several tumors and among them GBM (7). Autophagy is differently modulated by environmental conditions: EGFR inhibits and promotes autophagy in nutrient-rich conditions and under starvation, respectively. This latter activity correlates with cancer cell resistance and survival. Cells cultures under nutrient-rich conditions showed that EGFRvIII expression is rapidly lost (8). Expression of EGFRvIII increases the activation of autophagy during starvation and hypoxia, both of which can realize in the heterogeneous tumor microenvironment, thus providing a survival advantage. Through intracellular degradation of macro-components such as damaged organelles and proteins, cancer cells under stress conditions can rapidly obtain multiple substrates for cell metabolism. EGFR regulates the intracellular trafficking of subcellular organelles, like mitochondria. Under threatening conditions for cancer cells, like the presence of EGFR inhibitors and apoptosis inducers, EGFR translocates in mitochondria and contributes to drug resistance (9). EGFR and EGFRvIII exert anti-apoptotic functions through further mechanisms involving endoplasmic reticulum (ER)-residing protein, Reticulocalbin 1 (RCN1). RCN1 levels correlate with expression of EGFRvIII and overexpression of wt EGFR and contribute to cell survival under pathophysiological conditions that lead to endoplasmic reticulum stress (10). This recent finding represents further proof of the strict relationship among oncogenic pathways, microenvironmental conditions, and intracellular organelles. More directly, EGFR signaling profoundly influences cancer cell metabolism and is involved in the biosynthesis of fatty acids and pyrimidines until glucose catabolism. Strikingly, activated EGFR signaling fosters aerobic glycolysis which is included among the so-called non-canonical functions played by EGFR signaling (6). The physical connection of EGFR with sodium-glucose cotransporter 1 (SGLT1) increases the glucose influx. Moreover, EGFR controls two key glycolytic enzymes, that is hexokinase and pyruvate kinase. EGFR signaling also interferes with glutamine metabolism (11).

Looking to downstream pathways, oncogenic ras was proven to drive cancer cells toward an anabolic metabolism with the results to increase biomass production and support unconstrained proliferation (12). The previously mentioned autophagy mechanism was shown to be increased in ras-driven tumors (13). Oncogenic Kras shunted glucose-derived metabolites to the non-oxidative arm of the pentose phosphate pathway (PPP) to increase nucleic acid production (14).

The PI3K-Akt-mTOR pathway controls the uptake and use of several nutrients with the primary aim to support enhanced cancer cell proliferation. This signaling network exerts control over nutrient transporters and metabolic enzymes, thus playing a key role in cellular metabolism (15). The activity of the PI3K-Akt-mTOR pathway is normally counteracted by PTEN which, however, is frequently inactivated in GBM. mTOR supervises control of protein synthesis and cell cycle entry by the complex mTORC1 and interferes with cell metabolism and aerobic glycolysis with the mTORC2 complex. Specifically, mTOR through mTORC2 induces tight dependence on glucose. PTEN is not only a tumor suppressor. It acts as a regulator of mitochondrial metabolism and autophagy (16), governs different metabolic processes like glycolysis, gluconeogenesis, glycogen synthesis, as well as lipid metabolism (17) and reduces the level of phosphatidylinositol-3, 4, 5-phosphate (PIP3), a critical 2nd messenger of growth factors and insulin. Metabolic signals by PTEN are particularly relevant in metabolic tissues like the liver, adipose tissue, and muscle. Recently, the role of PTEN in opposition to cancer cell metabolic reprogramming was highlighted. PTEN reduces glucose influx and addresses cells to the mitochondrial Krebs cycle (“anti-Warburg state”), differently from cancer cells that privilege anaerobic glycolysis (“Warburg state,” see later) (16).

p53 is the most mutated gene in human cancers. The tumor suppression activity is tightly maintained through different mechanisms in cells, including ubiquitination and degradation which involves MDM2. The tumor suppressor and transcription factor p53 is considered as a cell guardian with the role of a supervisor in DNA damages, cell death, and cell cycle control. Recent studies outline the role of p53 in the regulation of various metabolic pathways. p53 acts as a negative regulator of glycolysis. This action starts at the level of the cell membrane through repression of the SGLT1 and SGLT4 transcription (18) and proceeds to downregulation of protein levels of hexokinase 2. Moreover, p53 is associated with inhibition of the pentose phosphate pathway through the negative regulation of glucose 6-phosphate dehydrogenase (G6PD), thus reducing nucleotide synthesis. Loss of p53 function contributes to addressing cancer cells to a Warburg-oriented metabolism.

Finally, also the Rb pathway has not only a defined role in the cell cycle but also regulates glucose tolerance, and the expression of genes involved in central carbon metabolism.

Apart from genetic pathways, a relevant role in heterogeneity and survival of GBM is played by glioma stem cells (GSCs). GSCs are physically located in niches near vessels and thus can easily survive compared to cells in necrotic areas. They retain the ability to maintain cells belonging to their lineage, can differentiate or undergo epithelial to mesenchymal transition (EMT) displaying more aggressive behavior. GSCs are metabolically dependent on oxidative phosphorylation, however, they can use additional metabolic pathways under stress conditions. This implies that targeting glycolysis in glioma may spare GSCs. GSC represents a core even more refractory to traditional treatments.

## Warburg Effect in Cancer and GBM Cells

Cancer cell studies have traditionally framed seven features that differentiate tumor from normal cells: (1) activated growth signal pathways; (2) apoptosis escape; (3) refractoriness to antigrowth signals; (4) uncontrolled proliferation; (5) angiogenesis; (6) invasion and metastasis; (7) aerobic glycolysis.

The altered tumor metabolism has long been acknowledged. Indeed, the basic difference between cancer cells from their normal counterpart is the potential for unconstrained proliferation, which requires a distinctive metabolic program. One key difference is the need to increase biomass, which is the primary need for a rapidly proliferating tumor cell. Tumor cells specialize to use diverse fuel sources to produce ATP as well as to maintain high levels of biosynthesis which can serve to promote survival in a progressively more adverse tumor microenvironment. Tumor cells must be able to adapt to low oxygen conditions and different or low nutrient availability. However, since the beginning of 1,900, cancer cells have been characterized by preferential use of aerobic glycolysis at expense of oxidative phosphorylation. The first process is characterized by high glucose uptake by cancer cells which enter an apparently inefficient pathway as compared to oxidative phosphorylation which realizes into mitochondria. Starting with one mole of glucose, aerobic glycolysis generates only 2 ATP molecules as compares to 36 ATP produced by mitochondrial phosphorylation. While glycolysis is preferred in the anaerobic conditions typical of tumor growth, the strange thing is that cancer cells privilege this metabolic pathway even in the presence of oxygen. This effect was named by its investigator as the Warburg effect. Tumor preference for aerobic glycolysis is probably based on the choice of a fast mechanism that is generally selected by rapidly proliferating normal cells, suggesting a potential advantage for growth derived also by the synthesis of biomass. The fact is that the enhanced influx of glucose within cancer cells produced 24 ATP molecules through aerobic glycolysis as compares to one ATP molecule generated by respiration (19). Moreover, it was supposed to arise from dysfunctional mitochondria in GBM, a currently obscure finding. Altered morphology compared to normal, abnormal bioenergetics as attested by the privileged aerobic glycolysis, significant alteration in the mitochondrial genome, and mutations in IDH have all been found in gliomas (20). Mitochondrial DNA depletion and mutations are associated with multidrug resistance (21). However, it is currently unclear if mitochondrial dysfunction represents a primary or a secondary event in gliomagenesis. The prevalent aerobic glycolysis is not a universal finding in GBM, in fact, glioma cell lines depending on mitochondrial oxidative phosphorylation have been identified (22).

While most of the studies have centered their attention on glucose metabolism for glioma cells, fatty acids biosynthesis and oxidation, as well as amino acid metabolism, are also relevant pathways for energy production and DNA synthesis. Once again, oncogenic pathways and lipid metabolism are interrelated. In GBM cellular models, inhibition of fatty acid synthase by inhibitors used for the treatment of obesity like orlistat significantly inhibits cell growth (23).

Glutamine metabolism represents another relevant way used by cancer cells to support protein synthesis and provide substrates to Kreb's cycle. The waste products of tumor cell metabolism, i.e., lactic, and succinic acid, contribute to acidify the tumor microenvironment, this increasing tumor progression.

The results of a century of investigations concerning this field in GBM can be summarized into the appreciation of the cancer cell's abilities to use all metabolic pathways according to extracellular variations. A persistent intracellular background noise represents the dialogue among oncogenic and metabolic pathways and links different metabolic pathways to each other with impressive adaptability.

## Ketogenic Diet in GBM

Normal brain function requires a high amount of energy which represents 20% of the body's total energy at rest (24). This high energy request supports all cellular processes, maintenance of ion gradients, and synaptic transmission. As previously outlined, glucose is the favorite fuel for the brain and defective glucose metabolism is found in several neurodegenerative diseases which are characterized by glucose hypometabolism. On the other hand, glucose is avidly captured by glioma cells to support the even increased metabolism and becomes a potential poison for GBM patients by increasing cancer proliferation. Apart from the previously highlighted mechanisms, glucose increases insulin and insulin-like growth factors which can further function as cancer promoters. The frequent steroids use in the management of brain tumors also contributes to feeding vicious circles related to glucose metabolism. There is contradictory evidence concerning the relevance of hyperglycemia and diabetes mellitus in GBM. While some studies did not find a correlation with overall and progression-free survival (25–27), others outline that hyperglycemia and elevated Body Mass Index are independent risk factors for poor outcome (28, 29). Moreover, all standard treatments used in GBM may paradoxically contribute to facilitating tumor growth, not only increasing blood glucose but also glutamate levels. This latter is an excitatory neurotransmitter promoting tumor invasion by itself and contributing to tumor energy requirement through conversion into glutamine.

When glucose supply is reduced, ketone bodies, i.e., acetoacetate and beta-hydroxybutyrate, produced in the liver, reach the brain, cross the blood-brain barrier through monocarboxylate transporters (MCTs), and together with lactate suit to the brain energetic metabolism. However, while normal cells can use ketone bodies for their own energetic needs, tumor cells lacking functional mitochondria are simultaneously deprived of the preferred substrate, that is glucose, and incompetent to use the alternative source represented by ketones. MCTs and the chaperone CD147 rule glucose cell uptake and lactate efflux, thus maintaining pH homeostasis and participating in the Warburg effect. Tumor cells overexpressing CD147 gain an advantage from a metabolic point of view and enhance their invasive and metastatic potential (30). These rationales and the high expression levels of CD147 in several kinds of tumors including GBM (31) could have therapeutic implications.

Increasing evidence supports a role for ketone bodies in epigenetic modulation. As the Greek origin of the name suggests, epigenetics refers to subtle and editable modifications to DNA and histones that do not change the genotype. These modifications consist in methylation, preferentially acting as repressors of gene transcription, and acetylation producing activation of gene transcription. As an example of the relevance of epigenetic modulations, two different profiles of GBM can be differentiated according to the methylation profile (32). IDH1-mutant GBM, traditionally associated with a more favorable prognosis, showed hypermethylation in the promoter of different glycolytic enzymes with a subsequent reduction of transcription of these genes. On the contrary, mesenchymal GBMs are characterized by hypomethylation and following activation of transcription of glycolytic enzymes. Besides, the oncometabolite 2-hydroxyglutarate (2-HG) produced in mutant IDH tumors inhibits histone demethylases, inducing a hypermethylation status (32).

Additional changes in gene expression suggest that the ketogenic diet inhibits growth factors like insulin-like growth factor 1 (IGF-1), platelet-derived growth factor (PDGF), and epidermal growth factor receptor (EGFR) (33).

Increased plasma ketone levels are produced not only by fasting states but also by ketogenic diets and ingestion of supplements such as ketogenic medium-chain fatty acids (MCFA). The classic ketogenic diet is characterized by a ratio of 4:1 between fats and carbohydrates. Modified ketogenic diets provide a lower ratio of fats to carbohydrates equal to 3:1, 2:1, or 1:1. Loose variants of ketogenic diets are represented by modified Atkins diet (MAD), treatments based on a low glycemic index (LGIT), and a ketogenic diet supplemented with MCFA (34). Each privileged food in KD has a conceivable cell metabolic interference and all are claimed to produce increased energy and cognitive function improvements: as an example, fish contains high omega-3 acids levels with a recognized anti-inflammatory potential, eggs may contribute to a shift from a glycolytic to an oxidative phosphorylation based-metabolism through the high leucine levels (35). All the others ketogenic nutrients such as beef, Coconut oil, Greek yogurt, avocados, butter, low-carb vegetables, nuts, and seeds are high energy and/or antioxidant foods.

The beneficial effects of ketones on brain metabolism concern several aspects: enhanced mitochondrial biogenesis, antioxidant activity, epigenetic modulation of genes related to metabolism.

Animal models of glioma have shown that KD synergizes with radiotherapy (36), has anti-inflammatory properties, and reduces peri-tumoral edema and angiogenesis.

The first clinical evidence of a beneficial effect of KD in humans comes from the control of refractory seizures in children and adults (33, 37).

The KD is a widely known complementary adjunct to anticancer conventional treatments, however, its role remains controversial, and patients' adherence is limited.

Several limits can be found in maintaining the strand of KD in GBM. The preclinical studies have intrinsic problems that reduce the value of obtained results. As examples, initial cultured cells underwent epigenetic changes in the presence of serum, and xenografts of human glioma cells were implanted into the flanks of animals and used in immunocompromised mice (38). All these models are intuitively unable to reproduce *in-vivo* biology and are far away from tumor heterogeneity and the complex glioma microenvironment.

Considering the mole of data supporting calorie restriction and KD, recent data identifies an opposite picture: the unrestricted KD has not always stopped tumor growth (39). A recent study showed that U87 glioma cell line and patient-derived GBM cultures utilize fatty acids and ketones for growth (40). Given these results, KD cannot be uniformly considered as an anti-cancer adjuvant treatment but can become a pro-growth factor. This finding is a warning toward an unconditioned adhesion to rigorous KD.

Rigorous protocols of KD require frequent blood glucose and ketone monitoring. KD can be maintained for limited periods and all studies documented a time-related clinical effect that dissolves when KD is stopped. Most importantly, the achievement of adequate ketosis is usually measured with ketone and glucose blood levels and their ratio (Glucose Ketone Index). However, these measures do not reflect ketone levels in the brain notwithstanding that the level of brain ketosis required to achieve significant metabolic and perhaps the antitumor effect is not known. Some studies used MR spectroscopy to evaluate cerebral ketones in patients with high-grade glioma during a KD (41). These trials open the way to radiomics application in this setting. Adherence to KD is often poor and potentially reduces patients' and family's quality of life. Adverse events can also be observed such as weight loss, which sometimes is not desired, gastrointestinal problems, increased levels of blood lipids, deficiency in vitamins and minerals. Finally, all published studies present several limitations. A small number of patients were enrolled, and feasibility was the primary endpoint. This means that no definitive conclusion on clinical activity can be drawn. ERGO trial evaluated the feasibility of an unrestricted ketogenic diet in 20 patients with recurrent GBM (42). In this study, the clinical activity of KD was considered as “moderate” at best. highlighting that the used protocol did not produce low glucose levels, that might be related to steroid use and lack of calorie restriction. ERGO2 trial evaluates a calorically restricted KD and intermittent fasting (KD-IF) in addition to reirradiation for recurrent malignant gliomas. In the MR-spectroscopic part of this trial, tumors were shown to generate ATP using alternative energy sources when there are low serum glucose levels (43). This finding can be added to the evidence against KD.

Schwartz et al., in the study published in 2018, enrolled 15 patients (44) administering a ketogenic 3:1 diet as an addition to standard treatments. Limited information on clinical activity was available. The recently published Keating study evaluates the ketogenic diet as an adjuvant to standard therapy in 12 GBM patients which were randomized to a modified ketogenic diet (MKD) or medium-chain triglyceride ketogenic diet (MCTKD) (45). Once again, the scientific relevance of this trial is questionable given that only 4 patients completed the 3-month diet. Recently, attempts to prolong the time on KD and to standardize diet protocols were performed (46, 47). The longest studies apply a 14- and 24-week duration of KD. This latter used a 1,600 Kcal/day total meal replacement program. However, once again, the small sample size reduces the relevance of the reports.

Recruiting studies on KD in GBM are summarized in Table 1. No phase 3 and only one phase 2 trial are reported. Another proof of the relevant role of metabolism in brain tumors and the search for effective modulatory strategies can be found in the NCT04691960 study. As reported in Table 1, this trial includes metformin because of its known hypoglycemic effects and potential anticancer activity. Despite the use of an antidiabetic drug as an adjunct to anticancer therapies is still debated, several studies support a relevant role in several tumors.

**Table 1:** Recruiting studies on KD in GBM.

**Clinical trials**	**Type of study**	**Study start date/estimated study completion date**	**Participants**	**Diet**	**Notes**	**Diet duration**	**Primary endpoint**
NCT04691960	Phase 2	August 2016/December 2024	36	KD 3:1; 4:1 if ketosis is not achieved	Metformin	Continuous (average: 8 months)	Feasibility
NCT03451799IIT2016-17-HU-KETORADTMZ	Phase 1	April 2018/April 2021	20	KD		16 weeks	Safety
NCT03278249	Not applicable	October 2017/January 2021	30	Modified Atkins Ketogenic Diet	<20 g of carbohydrates per day	6 months	Assessment of inducing ketosis

*Source: clinicaltrials.gov (accessed April 11, 2021)*.

The use of metformin is encouraged by data coming from preclinical and clinical studies. Metformin can inhibit the signal transducer and activator of transcription number 3 (STAT3) which is an important pathogenetic factor in GBM through the effect on brain tumor-initiating cells (BTICs) and differentiated cells (48). Interestingly, the gene signature typically induced by the hypoxic GBM microenvironment was partially modified by metformin in a cell model (49). A recent systematic review showed that the combination of metformin with temozolomide given post-radiotherapy achieved better OS and PFS as compares with temozolomide alone (50). However, a recent pooled analysis produced opposite results (26).

## Gut Microbiome, Gut-Brain Axis, and KD: A Broadband COnnection Still to Explore

Human gut microbiota contains around 10^13^-10^14^ microbes belonging to more than one thousand species which represents a significant genetic pool. The gut microbiota significantly contributes to the maturation of the gut immune system (51). Apart from systemic immune regulation, the microbiome has emerged to be involved also in neurophysiology and microglia development.

Several studies have documented a role for the microbiome in certain tumors. Excluding more linked sites such as the colon and liver, an increasing amount of evidence supports the so-called “gut-brain axis” which summarizes all the routes of reciprocal interferences between these systems. Communication is founded on messages transmitted through blood, lymphatics, and nerve fibers, i.e., the vagus nerve. These messages embodied into peptides released from enteroendocrine cells, neural transmitters, and immune cells run everywhere and also reach the brain.

Looking specifically to glioma, antibiotic treatment through a gut microbiome-immune cells-microglia circuit oriented to a pro-inflammatory pathway leads to increased glioma growth in mice models (52). Moreover, a different gut microbiome diversity was shown in mice and glioma patients as compared with healthy subjects and temozolomide was able to restore the pattern found in physiological conditions (53, 54).

The different populations of gut microbes can be considered beneficial or potentially dangerous to the host.

Short-chain fatty acids (SCFAs), predominantly consisting of acetate, propionate, and butyrate, are almost exclusively derived from bacterial metabolism in the gut and have been implicated in a variety of physiological processes and (neuro) immune functions. Gut microbiota influences immune responses through SCFAs that reduce proinflammatory cytokines and contribute to Treg development (55). Recently, glioma growth was shown to decrease SCFAs in the fecal composition in mice, while this effect was not observed during temozolomide treatment (56). In this study, a decrease in Bacteroides and Firmicutes phyla levels, an increase in Verrucomicrobia phylum, Akkermansia, and Bacteroides genera were found after tumor growth in mice.

Gut microbiota can influence cancer by alteration of immune responses, response to treatment, and direct notching host DNA through genotoxins. Metabolites formed by the microbiome can produce epigenetic changes (57). Recent investigations are disclosing intriguing circuits that involve neurotransmitters produced by the gut microbiome with an effect on cells at a different stage of differentiation (58).

Since the colonization of the gut at birth, the microbiome is influenced by external factors like mode of delivery and breastfeeding. Thereafter, the most influential recognized factors are diet and drugs such as antibiotics.

Nutritional habits are related to the differential growth of gut microbes. While a high intake of non-refined foods and fibers support the growth of microbes specialized in the production of SCFAs, a high fat/high sugar and low fibers intake, typical of the Western diet, promotes the production of detrimental metabolites, and decrease SCFAs, favoring the expansion of bacteria associated with chronic inflammation (59). In mice fed with ketogenic diets, two species of putatively beneficial bacteria, Akkermansia and Parabacteriodes, significantly increased compared to potentially harmful phyla (59, 60). These shreds of evidence support the gut microbiota remodeling into a “keto microbiota” (61) which can result as a useful adjuvant to standard treatments. Specialized and restricted dietary regimens may affect positively or negatively the microbiota composition and, therefore, influence host physiology and disease evolution and outcome. Several studies investigated the effect of diet on neurological disorders, from epilepsy (62) to neurodegenerative diseases (63). Different kinds of diet are associated with a different gut microbiota signature, immune response, and effect on the mucus intestinal layer (64). However, some concerns on the effects of KD on the gut microbiota have been raised especially as regards damaged mucus layer homeostasis and reduced total bacteria abundance and diversity (64).

## Discussion

The actual outlook as concerns clinical ongoing trials suggests that interest in KD seems exhausted. However, definitive conclusions cannot be achieved.

As in other tumors, insights into the different subtypes may provide a key to interpreting the multifaceted aspects of a given cancer. In the case of GBM, a recent classification derived from a computational analysis identifies four subgroups of GBM: proliferative/progenitor, neuronal, mitochondrial, and glycolytic/plurimetabolic (65). Strikingly, from this classification emerge that oxidative phosphorylation is the unique metabolic way of energy production used by the mitochondrial subtype which is characterized by a more favorable clinical outcome. This subtype could benefit from targeted metabolic therapies, such as inhibitors of mitochondrial metabolism. On the other hand, the poor-prognosis, glycolytic/plurimetabolic subgroup use multiple cell energy-producing programs, thus, a clear Achilles heel is not clearly detectable. This latter subtype could be more susceptible to metabolic interventions. Focusing on these two subtypes, the two-faced Janus of GBM is represented by a 20% of GBM with overactive mitochondria and the plurimetabolic subtype that mostly matched to a tumor characterized by inactivation of mitochondria and dominant Warburg effect. This characterization opens the way for a tailored defined approach to GBM that could include subtype selection and comprehensively investigate peripheral (gut), biochemical and central effects of any given intervention. This kind of approach is typical of integrative medicine that contemplates standard treatment associated with complementary medicine (66–68).

Metabolic reprogramming of GBM is a fascinating story that merits further well-designed investigations taking into account the increased knowledge on the multiple broad interferences and GBM diversity. The Gut-brain axis breaks down any concept on physical barriers and carries the message that extensive studies are needed to define the variegate interplay among metabolic and signaling pathways. Our feeling is that the urgent need for clinical opportunities overmatches the incomplete preclinical definition of this matter and only advancements in both sides could guide the future perspectives in this field.

## Author Contributions

LM had the idea and wrote the paper. FS edited Figure 1. LA provided points of reflection and contributed to improving the manuscript. VC, LS, SDC, SDS, AM, BF, FD, MB, RF, and GF critically revised the paper. All authors have approved the final version.

## Conflict of Interest

The authors declare that the research was conducted in the absence of any commercial or financial relationships that could be construed as a potential conflict of interest.

## Footnotes

^1^https://clinicaltrials.gov/ (accessed April 6, 2021).

## References

[1] Stupp R Mason WP van den Bent MJ Weller M Fisher B Taphoorn MJB. European organisation for research and treatment of cancer brain tumor and radiotherapy groups; national cancer institute of canada clinical trials group. Radiotherapy plus concomitant and adjuvant temozolomide for glioblastoma. N Engl J Med. (2005) 352:987–96. 10.1056/NEJMoa043330 15758009

[2] Alnahhas I Alsawas M Rayi A Palmer JD Raval R Ong S. Characterizing benefit from temozolomide in MGMT promoter unmethylated and methylated glioblastoma: a systematic review and meta-analysis. Neurooncol Adv. (2020) 2:vdaa082. 10.1093/noajnl/vdaa082 33150334 PMC7596890

[3] Puduvalli VK Chaudhary R McClugage SG Markert J. Beyond alkylating agents for gliomas: quo vadimus? Am Soc Clin Oncol Educ Book. (2017) 37:175–86. 10.1200/EDBK_175003 28561663 PMC5803081

[4] Oprita A Baloi S-C Staicu G-A Alexandru O Tache DE Danoiu S. Updated Insights on EGFR signaling pathways in glioma. Int J Mol Sci. (2021) 22:587. 10.3390/ijms22020587 33435537 PMC7827907

[5] Lombardi MY Assem M De Vleeschouwer S. Chapter 1: Glioblastoma Genomics: a very complicated story. In: De Vleeschouwer S, editor. Glioblastoma. Brisbane, QLD: Codon Publications (2017). 29251861

[6] Sigismund S Avanzato D Lanzetti L. Emerging functions of the EGFR in cancer. Mol Oncol. (2018) 12:3–20. 10.1002/1878-0261.12155 29124875 PMC5748484

[7] Rodrigues T Ferraz LS. Therapeutic potential of targeting mitochondrial dynamics in cancer. Biochem Pharmacol. (2020) 182:114282. 10.1016/j.bcp.2020.114282 33058754

[8] Bigner SH Humphrey PA Wong AJ Vogelstein B Mark J Friedman HS. Characterization of the epidermal growth factor receptor in human glioma cell lines and xenografts. Cancer Res. (1990) 50:8017–22. 2253244

[9] Cao X Zhu H Ali-Osman F Lo HW. EGFR and EGFRvIII undergo stress- and EGFR kinase inhibitor-induced mitochondrial translocalization: a potential mechanism of EGFR-driven antagonism of apoptosis. Mol Cancer. (2011) 10:26. 10.1186/1476-4598-10-26 21388543 PMC3063231

[10] Gomez J Areeb Z Stuart SF Nguyen HPT Paradiso L Zulkifli A. EGFRvIII promotes cell survival during endoplasmic reticulum stress through a reticulocalbin 1-dependent mechanism. Cancers (Basel). (2021) 13:1198. 10.3390/cancers13061198 33801941 PMC7999088

[11] Yang R Li X Wu Y Zhang G Liu X Li Y. EGFR activates GDH1 transcription to promote glutamine metabolism through MEK/ERK/ELK1 pathway in glioblastoma. Oncogene. (2020) 39:2975–86. 10.1038/s41388-020-1199-2 32034306

[12] Alec C. Kimmelman metabolic dependencies in RAS-driven cancers. Clin Cancer Res. (2015) 21:1828–34. 10.1158/1078-0432.CCR-14-2425 25878364 PMC4400826

[13] Kim MJ Woo SJ Yoon CH Lee JS An S Choi YH. Involvement of autophagy in oncogenic K-Ras-induced malignant cell transformation. J Biol Chem. (2011) 286:12924–32. 10.1074/jbc.M110.138958 21300795 PMC3075639

[14] Ying H Kimmelman AC Lyssiotis CA Hua S Chu GC Fletcher-Sananikone E. Oncogenic Kras maintains pancreatic tumors through regulation of anabolic glucose metabolism. Cell. (2012) 149:656–70. 10.1016/j.cell.2012.01.058 22541435 PMC3472002

[15] Hoxhaj G Brendan D. The PI3K-AKT network at the interface of oncogenic signalling and cancer metabolism. Nat Rev Cancer. (2020) 20:74–88. 10.1038/s41568-019-0216-7 31686003 PMC7314312

[16] Aquila S Santoro M Caputo A Panno ML Pezzi V De Amicis F. The tumor suppressor PTEN as molecular switch node regulating cell metabolism and autophagy: implications in immune system and tumor microenvironment. Cells. (2020) 9:1725. 10.3390/cells9071725 32708484 PMC7408239

[17] Chen C-Y Chen J He L Stiles BL. PTEN: tumor suppressor and metabolic regulator. Front Endocrinol (Lausanne). (2018) 9:338. 10.3389/fendo.2018.00338 30038596 PMC6046409

[18] Liu J Zhang C Hu W Feng Z. Tumor suppressor p53 and metabolism. J Mol Cell Biol. (2019) 11:284–92. 10.1093/jmcb/mjy070 30500901 PMC6487777

[19] Pfeiffer T Schuster S Bonhoeffer S. Cooperation and competition in the evolution of ATP-producing pathways. Science. (2001) 292:504–7. 10.1126/science.1058079 11283355

[20] Guntuku L Naidu VGM Yerra VG. Mitochondrial dysfunction in gliomas: pharmacotherapeutic potential of natural compounds. Curr Neuropharmacol. (2016) 14:567–83. 10.2174/1570159X14666160121115641 26791479 PMC4981742

[21] Pascale RM Calvisi DF Simile MM Feo CF Feo F. The warburg effect 97 years after its discovery. Cancers. (2020) 12:2819. 10.3390/cancers12102819 33008042 PMC7599761

[22] Libby CJ Tran AN Scott SE Griguer C Hjelmeland AB. The pro-tumorigenic effects of metabolic alterations in glioblastoma including brain tumor initiating cells. Biochim Biophys Acta Rev Cancer. (2018) 1869:175–88. 10.1016/j.bbcan.2018.01.004 29378228 PMC6596418

[23] Grube S Dünisch P Freitag D Klausnitzer M Sakr Y Walter J. Overexpression of fatty acid synthase in human gliomas correlates with the WHO tumor grade and inhibition with Orlistat reduces cell viability and triggers apoptosis. J Neurooncol. (2014) 118:277–87. 10.1007/s11060-014-1452-z 24789255

[24] Jensen HJ Wodschow HZ Nilsson M Rungby J. Effects of ketone bodies on brain metabolism and function in neurodegenerative diseases. Int J Mol Sci. (2020) 21:8767. 10.3390/ijms21228767 33233502 PMC7699472

[25] Barami K Lyon L Conell C. Type 2 diabetes mellitus and glioblastoma multiforme-assessing risk and survival: results of a large retrospective study and systematic review of the literature. World Neurosurg. (2017) 106:300–7. 10.1016/j.wneu.2017.06.164 28698089

[26] Seliger C Genbrugge E Gorlia T Chinot O Stupp R Nabors B.; EORTC brain tumor group. Use of metformin and outcome of patients with newly diagnosed glioblastoma: pooled analysis. Int J Cancer. (2020) 146:803–9. 10.1002/ijc.32337 30980539

[27] Disney-Hogg L Sud A Law PJ Cornish AJ Kinnersley B Ostrom QT. Influence of obesity-related risk factors in the aetiology of glioma. Br J Cancer. (2018) 118:1020–7. 10.1038/s41416-018-0009-x 29531326 PMC5931112

[28] Lu VM Goyal A Vaughan LS McDonald KL. The impact of hyperglycemia on survival in glioblastoma: a systematic review and meta-analysis. Clin Neurol Neurosurg. (2018) 170:165–9. 10.1016/j.clineuro.2018.05.020 29803727

[29] Montemurro N Perrini P Rapone B. Clinical risk and overall survival in patients with diabetes mellitus, hyperglycemia and glioblastoma multiforme. A review of the current literature. Int J Environ Res Public Health. (2020) 17:8501. 10.3390/ijerph17228501 33212778 PMC7698156

[30] Li X Yu X Dai D Song X Xu W. The altered glucose metabolism in tumor and a tumor acidic microenvironment associated with extracellular matrix metalloproteinase inducer and monocarboxylate transporters. Oncotarget. (2016) 7:23141–55. 10.18632/oncotarget.8153 27009812 PMC5029616

[31] Riethdorf S Reimers N Assmann V Kornfeld JW Terracciano L Sauter G. High incidence of EMMPRIN expression in human tumors. Int J Cancer. (2006) 119:1800–10. 10.1002/ijc.22062 16721788

[32] Dong Z Cui H. Epigenetic modulation of metabolism in glioblastoma. Semin Cancer Biol. (2019) 57:45–51. 10.1016/j.semcancer.2018.09.002 30205139

[33] Woolf EC Scheck AC. Thematic review series: calorie restriction and ketogenic diets the ketogenic diet for the treatment of malignant glioma. J Lipid Res. (2015) 56:5–10. 10.1194/jlr.R046797 24503133 PMC4274070

[34] McDonald TJW Cervenka MC. The expanding role of ketogenic diet in adult neurological disorders. Brain Sci. (2018) 8:148. 10.3390/brainsci8080148 30096755 PMC6119973

[35] Viana LR Tobar N Busanello ENB Marques AC de Oliveira AG Lima TI. Leucine-rich diet induces a shift in tumour metabolism from glycolytic towards oxidative phosphorylation, reducing glucose consumption and metastasis in Walker-256 tumour-bearing rats. Sci Rep. (2019) 9:15529. 10.1038/s41598-019-52112-w 31664147 PMC6820796

[36] Abdelwahab MG Fenton KE Preul MC Rho JM Lynch A Stafford P. The ketogenic diet is an effective adjuvant to radiation therapy for the treatment of malignant glioma. PLoS ONE. (2012) 7:e36197. 10.1371/journal.pone.0036197 22563484 PMC3341352

[37] Barañano KW Hartman AL. The ketogenic diet: uses in epilepsy and other neurologic illnesses. Curr Treat Options Neurol. (2008) 10:410–9. 10.1007/s11940-008-0043-8 18990309 PMC2898565

[38] Strickland M Stoll EA. Metabolic reprogramming in glioma. Front Cell Dev Biol. (2017) 5:43. 10.3389/fcell.2017.00043 28491867 PMC5405080

[39] De Feyter HM Behar KL Rao JU Madden-Hennessey K Ip KL Hyder F. A ketogenic diet increases transport and oxidation of ketone bodies in RG2 and 9L gliomas without affecting tumor growth. Neuro Oncol. (2016) 18:1079–87. 10.1093/neuonc/now088 27142056 PMC4933488

[40] Sperry J Condro MC Guo L Braas D Vanderveer-Harris N Kim KKO. Glioblastoma utilizes fatty acids and ketone bodies for growth allowing progression during ketogenic diet therapy. iScience. (2020) 23:101453. 10.1016/j.isci.2020.101453 32861192 PMC7471621

[41] Berrington A Schreck KC Barron BJ Blair L Lin DDM Hartman AL. Cerebral ketones detected by 3T MR spectroscopy in patients with high-grade glioma on an atkins-based diet. AJNR Am J Neuroradiol. (2019) 40:1908–15. 10.3174/ajnr.A6287 31649157 PMC6856437

[42] Rieger J Bahr O Maurer GD Hattingen E Franz K Brucker D. ERGO: a pilot study of ketogenic diet in recurrent glioblastoma. Int J Oncol. (2014) 44:1843–52. 10.3892/ijo.2014.2382 24728273 PMC4063533

[43] Wenger KJ Wagner M Harter PN Franz K Bojunga J Fokas E. Maintenance of energy homeostasis during calorically restricted ketogenic diet and fasting-MR-spectroscopic insights from the ERGO2 trial. Cancers (Basel). (2020) 12:3549. 10.3390/cancers12123549 33261052 PMC7760797

[44] Schwartz KA Noel M Nikolai M Chang HT. Investigating the ketogenic diet as treatment for primary aggressive brain cancer: challenges and lessons learned. Front Nutr. (2018) 5:11. 10.3389/fnut.2018.00011 29536011 PMC5834833

[45] Martin-McGill KJ Marson AG Smith CT Young B Mills SJ Cherry MG. Ketogenic diets as an adjuvant therapy for glioblastoma (KEATING): a randomized, mixed methods, feasibility study. J Neurooncol. (2020) 147:213–27. 10.1007/s11060-020-03417-8 32036576 PMC7076054

[46] Klein P Tyrilova I Zuccoli G Tyrlik A Maroon JC. Treatment of glioblastoma multiforme with “classic” 4:1 ketogenic diet total meal replacement. Cancer Metab. (2020) 8:24. 10.1186/s40170-020-00230-9 33292598 PMC7653752

[47] Van der Louw EJTM Olieman JF van den Bemt PMLA Bromberg JEC Oomen-de Hoop E Neuteboom RF. Ketogenic diet treatment as adjuvant to standard treatment of glioblastoma multiforme: a feasibility and safety study. Ther Adv Med Oncol. (2019) 11:1–13. 10.1177/1758835919882584 31258628 PMC6589986

[48] Leidgens V Proske J Rauer L Moeckel S Renner K Bogdahn U. Stattic and metformin inhibit brain tumor-initiating cells by reducing STAT3-phosphorylation. Oncotarget. (2017) 8:8250–63. 10.18632/oncotarget.14159 28030813 PMC5352398

[49] Calvo Tardón M Marinari E Migliorini D Bes V Tankov S Charrier E. An experimentally defined hypoxia gene signature in glioblastoma and its modulation by metformin. Biology (Basel). (2020) 9:264. 10.3390/biology9090264 32887267 PMC7563149

[50] Takhwifa F Aninditha T Setiawan H Sauriasari R. The potential of metformin as an antineoplastic in brain tumors: a systematic review. Heliyon. (2021) 7:e06558. 10.1016/j.heliyon.2021.e06558 33869859 PMC8044986

[51] Kamada N Seo S-U Grace CY Núñez G. Role of the gut microbiota in immunity and inflammatory disease. Nat Rev Immunol. (2013) 13:321–35. 10.1038/nri3430 23618829

[52] D'Alessandro G Antonangeli F Marrocco F Porzia A Lauro C Santoni A. Gut microbiota alterations affect glioma growth and innate immune cells involved in tumor immunosurveillance in mice. Eur J Immunol. (2020) 50:705–711. 10.1002/eji.201948354 32034922 PMC7216943

[53] Patrizz A Dono A Zorofchian S Hines G Takayasu T Husein N. Glioma and temozolomide induced alterations in gut microbiome. Sci Rep. (2020) 10:21002. 10.1038/s41598-020-77919-w 33273497 PMC7713059

[54] Li X-C Wu B-S Jiang Y Li J Wang Z-F Ma C. Temozolomide-induced changes in gut microbial composition in a mouse model of brain glioma. Drug Design Dev Ther. (2021) 15 1641–52. 10.2147/DDDT.S298261 33907383 PMC8071088

[55] Mehrian-Shai R Reichardt JKV Harris CC Toren A. The gut-brain axis, paving the way to brain cancer. Trends Cancer. (2019) 5:200–7. 10.1016/j.trecan.2019.02.008 30961828 PMC6734924

[56] Dono A Patrizz A McCormack RM Putluri N Ganesh BP Kaur B. Glioma induced alterations in fecal short-chain fatty acids and neurotransmitters. CNS Oncol. (2020) 9:CNS57. 10.2217/cns-2020-0007 32602743 PMC7341178

[57] Krautkramer KA Kreznar JH Romano KA Vivas EI Barrett-Wilt GA Rabaglia ME. Diet-microbiota interactions mediate global epigenetic programming in multiple host tissues. Mol Cell. (2016) 64:982–92. 10.1016/j.molcel.2016.10.025 27889451 PMC5227652

[58] D'Alessandro G Lauro C Quaglio D Ghirga F Botta B Trettel F. Neuro-signals from gut microbiota: perspectives for brain glioma. Cancers. (2021) 13:2810. 10.3390/cancers13112810 34199968 PMC8200200

[59] Paoli A Mancin L Bianco A Thomas E Mota JF Piccini F. Ketogenic diet and microbiota: friends or enemies? Genes (Basel). (2019) 10:534. 10.3390/genes10070534 31311141 PMC6678592

[60] Ma D Wang AC Parikh I Green SJ Hoffman JD Chlipala G. Ketogenic diet enhances neurovascular function with altered gut microbiome in young healthy mice. Sci Rep. (2018) 8:6670. 10.1038/s41598-018-25190-5 29703936 PMC5923270

[61] Cabrera-Mulero A Tinahones A Bandera B Moreno-Indias I Macías-González M Tinahones FJ. Keto microbiota: a powerful contributor to host disease recovery. Rev Endocr Metab Disord. (2019) 20:415–25. 10.1007/s11154-019-09518-8 31720986 PMC6938789

[62] Olson CA Vuong HE Yano JM Liang QY Nusbaum DJ Hsiao EY. The gut microbiota mediates the anti-seizure effects of the ketogenic diet. Cell. (2018) 173:1728–41.e13. 10.1016/j.cell.2018.04.027 29804833 PMC6003870

[63] Rawat K Singh N Kumari P Saha L. A review on preventive role of ketogenic diet (KD) in CNS disorders from the gut microbiota perspective. Rev Neurosci. (2020) 32:143–57. 10.1515/revneuro-2020-0078 33070123

[64] Rinninella E Cintoni M Raoul P Lopetuso LR Scaldaferri F Pulcini G. Food components and dietary habits: keys for a healthy gut microbiota composition. Nutrients. (2019) 11:2393. 10.3390/nu11102393 31591348 PMC6835969

[65] Garofano L Migliozzi S Oh YT D'Angelo F Najac RD Ko A. Pathway-based classification of glioblastoma uncovers a mitochondrial subtype with therapeutic vulnerabilities. Nat Cancer. (2021) 2:141–56. 10.1038/s43018-020-00159-4 33681822 PMC7935068

[66] Inci H Inci F. Complementary and alternative medicine awareness in cancer patients receiving chemotherapy. WCRJ. (2020) 7:e1752. 10.32113/wcrj_202011_1752

[67] Berretta M Rinaldi L Taibi R Tralongo P Fulvi A Montesarchio V. Physician attitudes and perceptions of complementary and alternative medicine (CAM): a multicentre Italian study. Front Oncol. (2020) 10:594. 10.3389/fonc.2020.00594 32411599 PMC7202223

[68] Berretta M Della Pepa C Tralongo P Fulvi A Martellotta F Lleshi A. Use of complementary and alternative medicine (CAM) in cancer patients: an Italian multicenter survey. Oncotarget. (2017) 8:24401–414. 10.18632/oncotarget.14224 28212560 PMC5421857

